# Underestimation of Cardiovascular Risk by 2019 World Health Organization (WHO) Cardiovascular Risk Charts Compared With Framingham Global Risk Score (FGRS) in a Brazilian Population: Implications for Primary Prevention

**DOI:** 10.7759/cureus.104610

**Published:** 2026-03-03

**Authors:** Adalberto Teixeira da Matta Flora Neto, Lucas Petraglia Barroso, Leonardo Rodrigues Fernandes, João Lucas O'Connell

**Affiliations:** 1 Department of Cardiology, Universidade Federal de Uberlândia, Uberlândia, BRA

**Keywords:** cardiovascular diseases, damage prediction, heart disease risk factors, population forecast, world health organization

## Abstract

Introduction: Cardiovascular disease (CVD) remains the leading cause of mortality in Brazil. Accurate risk stratification is essential for guiding primary prevention, yet the concordance between global tools remains uncertain in specific populations. This study aimed to evaluate the agreement between the Framingham Global Risk Score (FGRS) and the 2019 World Health Organization (WHO) cardiovascular risk charts in a Brazilian population, assessing both calibration bias and the utility of non-laboratory models.

Methods: An observational, cross-sectional study was conducted at Hospital de Clínicas de Uberlândia (HC-UFU), a tertiary care center in Uberlândia, Brazil. A convenience sample of 140 adults (aged 40-74 years) was evaluated. Risk of 10-year cardiovascular events was estimated using the FGRS and WHO charts (Tropical Latin America region), applying both laboratory-based and non-laboratory (body mass index (BMI)-based) algorithms. Agreement was assessed using weighted kappa (kappa) and Bland-Altman analysis. We tested two threshold strategies: a standardized cut-off (≥20% for both) and tool-specific thresholds (FGRS ≥20% vs. WHO ≥10%) to assess clinical equivalence.

Results: The WHO charts systematically underestimated CVD risk compared to the FGRS. Using the standard ≥20% threshold, the FGRS identified 59.3% of men as high-risk, whereas WHO identified only 11.1%, revealing a substantial "prevention gap." Agreement between the FGRS and WHO was generally fair (kappa < 0.40) but improved significantly when a lower threshold (≥ 10%) was applied to the WHO charts. Conversely, internal consistency between laboratory and non-laboratory models was robust for both tools (kappa = 0.81), validating the use of BMI-based scores.

Conclusion: In this high-risk Brazilian population, WHO charts yielded significantly lower risk estimates than Framingham, potentially excluding eligible patients from statin therapy if standard thresholds are used. Adopting a lower treatment threshold (≥10%) for WHO charts may help achieve clinical equivalence with Framingham. Non-laboratory models demonstrated high reliability and offer a viable alternative for risk screening in resource-constrained settings.

## Introduction

Cardiovascular disease (CVD) remains the leading cause of mortality in low- and middle-income countries (LMICs), with age-standardized disability-adjusted life year (DALY) rates in lower sociodemographic regions nearly twice those observed in high-income settings [1]. In Brazil, CVD has been the leading cause of mortality since the 1960s, responsible for approximately 31% of all deaths [2]. This burden is particularly pronounced among populations with lower socioeconomic status and those with metabolic risk factors, such as hypertension and diabetes, which are rising in prevalence across Latin America [3,4]. Consequently, accurate risk stratification tools are essential for identifying high-risk individuals who would benefit most from primary prevention, particularly in resource-constrained settings where treatment allocation must be optimized [5].

The Framingham Risk Score (FRS), derived from predominantly Caucasian cohorts in the United States, has been widely adopted globally [6]. However, substantial evidence demonstrates that the original Framingham functions systematically overestimate absolute CVD risk when applied to external populations [6,7]. For instance, in comparative cohorts, predicted death rates often exceed actual rates significantly due to calibration bias, as the regression coefficients may not accurately reflect the magnitude of risk factor associations in ethnically and geographically diverse groups [6].

To address this, the World Health Organization (WHO) developed new risk prediction charts in 2019, calibrated to 21 global regions [5]. Designed to be practical for LMICs, these charts offer both laboratory-based and non-laboratory (body mass index (BMI)-based) versions. However, validation studies in Latin America have yielded conflicting results. While some studies suggest good discrimination, others document that WHO charts tend to classify significantly fewer individuals as "high-risk" compared to Framingham [8,9]. In one Eastern Caribbean study, the Framingham model identified 15 times more high-risk patients than the WHO model (31% vs. 2%) [9]. This potential for systematic underestimation raises concerns about treatment gaps in Brazilian primary care.

While global studies have suggested discrepancies between these tools, direct comparisons within the Brazilian high-risk context, specifically evaluating the non-laboratory WHO algorithms, remain scarce. We hypothesized that the WHO charts would systematically underestimate cardiovascular risk compared with Framingham in a high-risk Brazilian population, due to the lack of local validation for specific Brazilian metabolic clusters. Therefore, the primary objective of this study was to evaluate the concordance between the FGRS and the 2019 WHO cardiovascular risk charts in a high-risk Brazilian population to assess clinical equivalence as a proof-of-concept pilot investigation. Specifically, we aimed to quantify the agreement using both standardized and tool-specific thresholds, assess the systematic bias between the tools, and validate the internal consistency of the non-laboratory (BMI-based) models to support their use in low-resource settings.

## Materials and methods

This observational, cross-sectional study was conducted at the Hospital de Clínicas de Uberlândia (HC-UFU), Brazil, a tertiary care center, from July to December 2025. The setting was selected to evaluate a population with a high prevalence of metabolic risk factors. The protocol complied with the Declaration of Helsinki and the Brazilian National Health Council Resolution 466/2012. Approval was granted by the Institutional Ethics Committee (Opinion No. 7.675.760; CAAE: 86928125.0.0000.5152), and written informed consent was obtained from all participants.

A convenience sample of 140 adults was recruited consecutively from outpatient specialty clinics. The sample size was defined a priori based on the recommendation of 10 participants per variable for scale reliability analysis (the "10:1 rule")[10]. Consequently, the final sample of 140 participants exceeded the minimum requirement, ensuring sufficient statistical power for both internal consistency and correlation analyses. Inclusion criteria comprised patients of both sexes, aged 40 to 74 years, attending routine medical follow-ups, regardless of previous cardiovascular history or comorbidities. Exclusion criteria were limited to pregnancy, terminal illness, or missing clinical/laboratory data essential for risk score calculation. As this study was conducted in a tertiary hospital setting, the sample likely represents individuals with a higher cardiometabolic burden compared to the general population, which should be considered when interpreting the external applicability of the findings.

Participants underwent anthropometric assessment using a calibrated mechanical scale and a flexible inelastic tape for waist circumference. Blood pressure was assessed after five minutes of rest using a validated automatic oscillometric device, with the mean of two measurements used for analysis. Venous blood samples were collected after a 12-hour overnight fast. Serum concentrations of total cholesterol, HDL-cholesterol, triglycerides, and fasting glucose were determined by automated enzymatic colorimetric methods at the institution's central clinical laboratory.

Cardiovascular risk was estimated using the Framingham Global Risk Score (FGRS) [11] and the 2019 WHO Cardiovascular Risk Charts (Tropical Latin America region) [5], applying both laboratory-based and non-laboratory (BMI-based) algorithms. Both tools are open-access and validated for public use; therefore, specific copyright permission was not required. Agreement was evaluated using two approaches: a standardized threshold (≥20% for both), a strategy adopted in comparative literature [9,12], and guideline-recommended tool-specific thresholds (FGRS ≥ 20% vs. WHO ≥ 10%). Data analysis was performed using Jamovi software (The Jamovi Project (2025), retrieved from https://www.jamovi.org, Sydney, Australia). Continuous variables were presented as mean ± standard deviation (SD) or median (interquartile range), and categorical variables as frequencies. Agreement was assessed using the weighted kappa coefficient (kappa) and systematic bias via Bland-Altman plots. A p-value <0.05 was considered significant.

## Results

Clinical and sociodemographic characteristics of the sample

A total of 140 participants were included in the study, with a mean age of 59.1 ± 10.7 years. The sample showed a slight predominance of males (n = 81, 57.9%) compared to females (n = 59, 42.1%). Regarding the sociodemographic profile, a significant portion of the population presented low educational levels, with 37.1% (n = 52) classified as illiterate or having incomplete primary education (*x^2^* = 4.24, *p* = 0.237).

The clinical profile revealed a population with a high prevalence of metabolic risk factors. Metabolic syndrome was present in 76.4% (n = 107) of the participants (*x^2^* = 1.43, *p* = 0.232). Systemic arterial hypertension was the most prevalent known comorbidity (n = 101, 72.1%), followed by dyslipidemia (n = 77, 55.0%) and diabetes mellitus (n = 61, 43.6%). Regarding lifestyle factors, smoking history was reported by 45.0% (n = 63) of the sample, with a significantly higher prevalence among males than females (n = 44, 54.3% vs. n = 19, 32.2%; *x^2^* = 6.83, *p *= 0.009).

As detailed in Table 1, there were no statistically significant differences between sexes regarding age, BMI, waist circumference, or systolic and diastolic blood pressure levels. However, the lipid profile revealed distinct patterns: women exhibited significantly higher mean levels of total cholesterol (4.57 ± 1.29 mmol/L vs. 4.03 ± 1.20 mmol/L; *U* = 1835.0,* p* = 0.019) and HDL-cholesterol (1.18 ± 0.37 mmol/L vs. 1.05 ± 0.50 mmol/L; *U* = 1620.0, *p *= 0.001) compared to men.

**Table 1:** Baseline sociodemographic and clinical characteristics of the study population stratified by sex. Data are presented as mean ± standard deviation (SD) or frequency n (%). For continuous variables, independent t-test (*t*) or Mann-Whitney *U* test (*U*) were used based on normality. For categorical variables, the Chi-square test (*x^2^*) or Fisher's exact test was applied. A p-value < 0.05 was considered statistically significant. WC: waist circumference; DBP: diastolic blood pressure; SBP: systolic blood pressure; BMI: body mass index

Variables	Total (n = 140)	Males (n = 81)	Females (n = 59)	Test statistic	P-value
Demographics					
Age range (years), (mean ± SD)	59.05 ± 9.43	59.56 ± 8.89	58.42 ± 10.18	*U* = 2537.50	0.533
Education level, n (%)					
Illiterate / incomplete primary	52 (37.14%)	32 (39.51%)	20 (33.90%)	*x^2^* = 4.24	0.237
Primary education	31 (22.14%)	21 (25.93%)	10 (16.95%)		
Secondary education	48 (34.29%)	25 (30.86%)	23 (38.98%)		
Higher education	9 (6.43%)	3 (3.70%)	6 (10.17%)		
Smoking history, n (%)	63 (45%)	44 (54.32%)	19 (32.20%)	*x^2^* = 6.80	0.009
Known comorbidities / under treatment, n (%)					
Hypertension	101 (72.14%)	55 (67.90%)	46 (77.97%)	*x^2^* = 1.72	0.190
Diabetes	61 (43.57%)	35 (43.21%)	26 (44.07%)	*x^2 ^*= 0.01	0.919
Dyslipidemia	77 (55%)	42 (51.85%)	35 (59.32%)	*x^2 ^*= 0.77	0.380
Metabolic syndrome	107 (76.43%)	59 (72.84%)	48 (81.36%)	*x^2^* = 1.43	0.232
Clinical measurements (mean ± SD)					
WC (cm)	102.49 ± 18.85	101.85 ± 17.74	103.36 ± 20.39	*U* = 1976.0	0.690
DBP (mmHg)	81.06 ± 14.33	82.14 ± 15.88	79.54 ± 11.83	*t* = 1.07	0.285
SBP (mmHg)	128.53 ± 20.84	128.95 ± 21.51	127.95 ± 20.04	*t *= 0.28	0.780
HDL (mmol/L)	1.10 ± 0.45	1.05 ± 0.50	1.18 ± 0.37	*U *= 1620.0	0.001
Chol (mmol/L)	4.26 ± 1.26	4.03 ± 1.20	4.57 ± 1.29	*U *= 1835.0	0.019
BMI (kg/m²)	28.59 ± 7.64	27.53 ± 6.01	30.05 ± 9.28	*U *= 1976.0	0.081

Agreement analysis: the pitfalls of standardized thresholds

In the initial analysis, we compared the FGRS and WHO charts using standardized risk thresholds of 10% (moderate risk) and 20% (high risk) for both tools. Although these cut-offs align with FGRS definitions, they are often applied to WHO charts in comparative studies despite not being the standard intervals of the 2019 WHO manual.

**Figure 1:**
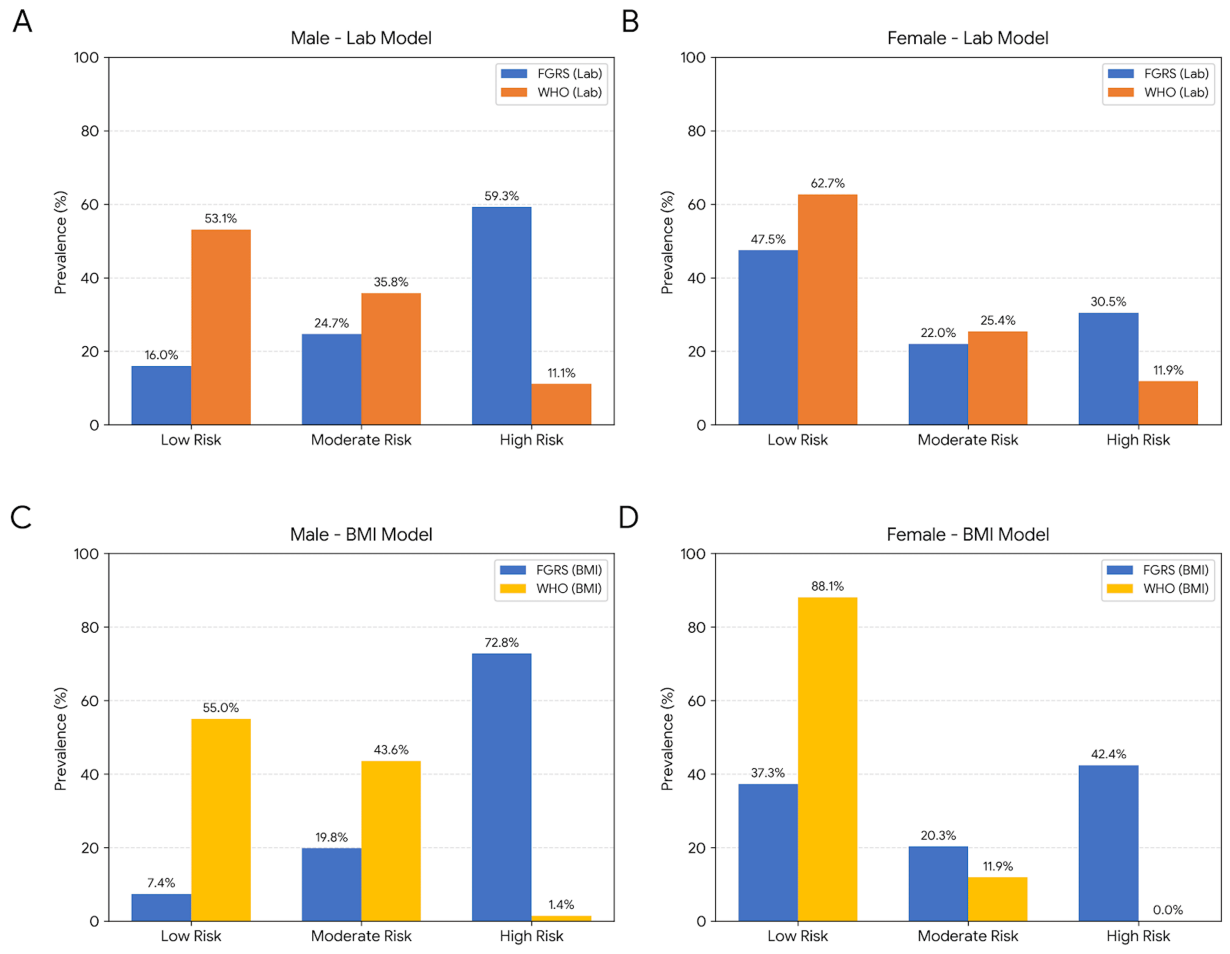
Comparison of risk categories using standardized thresholds (10% and 20%) for both tools. Note the extreme underestimation by the WHO charts when forced into the FGRS numerical criteria. (A) male laboratory model; (B) female laboratory model; (C) male BMI model; (D) female BMI model. Data are presented as frequencies (%). Total sample N = 140 (males n = 81; females n = 59). Significance level: p < 0.05. FGRS: Framingham Global Risk Score; WHO: World Health Organization Image generated using the Python programming language (v3.x), utilizing the Matplotlib and Seaborn libraries for high-resolution data visualization and statistical plotting

However, as shown in Figure 1, this standardization led to a significant discrepancy in risk classification, particularly among males. While the FGRS identified 59.3% of men as high risk (≥20%), the WHO laboratory model, when forced into the same threshold, identified only 11.1% (Figure 1A). An even more pronounced difference was observed in the BMI models, where the WHO chart identified a negligible 1.4% of high-risk individuals compared to 72.8% by the FGRS (Figure 1C). This poor clinical alignment was confirmed by the weighted kappa coefficients, which indicated only "fair" agreement for the male laboratory model (kappa = 0.29) and "slight" to "insignificant" agreement for the BMI-based comparisons (kappa < 0.10). Consequently, under the standard WHO criteria, a substantial proportion of high-risk patients identified by FGRS would be reclassified as low-to-moderate risk, potentially delaying necessary statin therapy.

**Figure 2:**
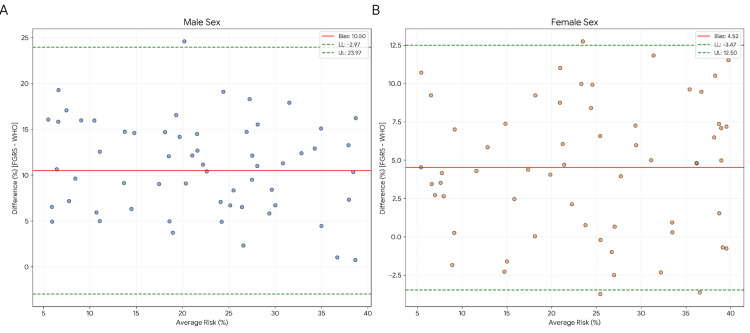
Bland-Altman plots representing the numerical discrepancy between FGRS and WHO absolute risk percentages. The plots demonstrate a significant systematic bias (A: +10.50% for males; B: +4.52% for females), demonstrating that the WHO charts consistently yield lower absolute risk values, which mathematically precludes the use of identical cut-off points for both tools. Data are represented as the difference in absolute risk percentage between models (bias) versus the average risk. The solid red line indicates the mean bias, and the dashed green lines represent the 95% limits of agreement (mean ± 1.96 SD). Total sample N = 140 (males n = 81; females n = 59). Statistical significance was set at p < 0.05. FGRS: Framingham Global Risk Score; WHO: World Health Organization Image generated using the Python programming language (v3.x), utilizing the Matplotlib and Seaborn libraries for high-resolution data visualization and statistical plotting

The statistical justification for this discrepancy is provided by the Bland-Altman plots (Figure 2). The analysis revealed a significant systematic bias of 10.50% for males and 4.52% for females. This indicates that the WHO charts consistently yield absolute risk percentages that are lower than those of the FGRS. Therefore, applying the same numerical thresholds (e.g., 20%) to both tools results in a severe underestimation of risk by the WHO charts, suggesting that these models are not directly interchangeable on a numerical scale.

Clinical agreement using tool-specific guidelines (WHO 2019 Criteria)

Following the identification of the systematic bias, a second analysis was conducted applying the tool-specific risk thresholds recommended for each tool's clinical application, rather than numerical equalization. For the FGRS, the standard classification was maintained: low risk (<10%), moderate risk (10-19%), and high risk (≥20%). For the WHO charts, considering the observed underestimation and adhering to the 2019 manual's stratification for low-resource settings, the thresholds were defined as: low risk (<5%), moderate risk (5-9%), and high risk (≥10%). Thus, the WHO ≥10% stratum was considered the clinical equivalent to the FGRS high-risk category for comparative purposes.

**Figure 3:**
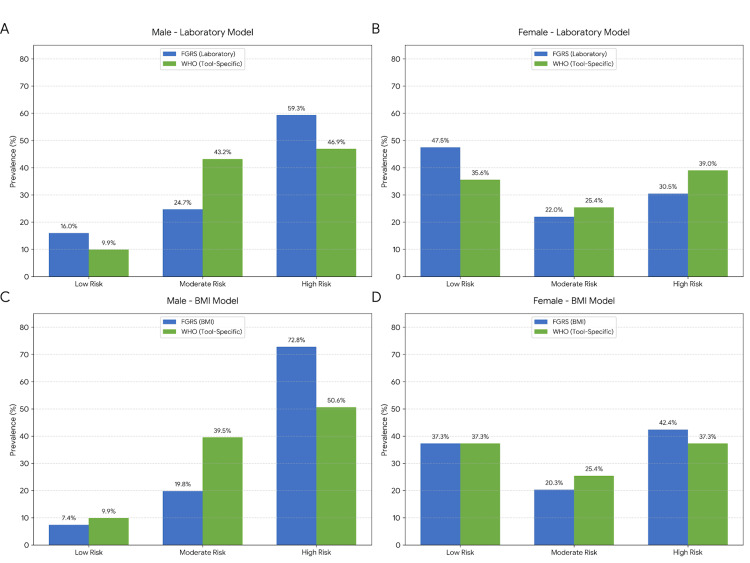
Cardiovascular risk stratification using tool-specific thresholds (WHO Manual Criteria). The comparison accounts for the distinct calibration between tools to define high risk: FGRS ≥ 20% versus WHO ≥ 10%. Note the substantial improvement in clinical agreement and the reduction in disparity compared to standardized thresholds (Figure 1). (A) male laboratory model; (B) female laboratory model; (C) male BMI model; (D) female BMI model. Green bars represent the WHO classification using the ≥ 10% cutoff. Data are presented as frequencies (%). Total sample N = 140 (males n = 81; females n = 59). Significance level: p < 0.05. FGRS: Framingham Global Risk Score; WHO: World Health Organization Image generated using the Python programming language (v3.x), utilizing the Matplotlib and Seaborn libraries for high-resolution data visualization and statistical plotting

Under these tool-specific calibrations, the risk stratification showed a marked convergence (Figure 3). In the male laboratory-based model (Figure 3A), the identification of high-risk individuals by the WHO charts rose significantly to 46.9%, reducing the disparity with the FGRS (59.3%). This adjustment improved the weighted kappa coefficient from 0.29 (fair) to 0.54 (moderate agreement). In the female population, the alignment was even more robust. The prevalence of high-risk women identified by the WHO charts (39.0%) closely matched the FGRS (30.5%) (Figure 3B), resulting in a weighted kappa of 0.87, classified as an "almost perfect" agreement.

A similar trend was observed in the non-laboratory (BMI) models. By applying the ≥10% threshold as the high-risk definition for the WHO BMI chart, the tool identified 50.6% of men and 37.3% of women as high risk, achieving Substantial agreement with the FGRS-BMI models (kappa = 0.63 for males; 0.81 for females), as detailed in Table 2.

**Table 2:** Comparison of weighted kappa coefficients for cardiovascular risk stratification under standardized versus tool-specific thresholds. Standardized thresholds: Comparison applying identical cut-off points (moderate: 10–19%; high: ≥20%) to both tools, disregarding the specific calibration of the WHO charts. Tool-specific thresholds: Comparison applying the specific cut-off points recommended by each tool's manual (FGRS high risk: ≥20% vs. WHO high risk: ≥10%). Kappa interpretation: <0.20 slight; 0.21–0.40 fair; 0.41–0.60 moderate; 0.61–0.80 substantial; 0.81–1.00 almost perfect. FGRS: Framingham Global Risk Score; WHO: World Health Organization; BMI: body mass index

Calibration strategy	Model type	Population	Weighted kappa (κ)	Agreement strength
Standardized thresholds (FGRS ≥20% vs. WHO ≥20%)	Laboratory	Male	0.29	Fair
		Female	0.75	Substantial
	BMI-based	Male	0.08	Slight
		Female	0.05	Slight
Tool-specific thresholds (FGRS ≥20% vs. WHO ≥10%)	Laboratory	Male	0.54	Moderate
		Female	0.87	Almost Perfect
	BMI-based	Male	0.63	Substantial
		Female	0.81	Substantial
Internal consistency	FGRS vs. WHO (BMI)	Male	0.63	Substantial
		Female	0.81	Substantial
	Lab vs. BMI (FGRS)	Total	0.81	Substantial
	Lab vs. BMI (WHO)	Total	0.60	Moderate

Agreement between laboratory and non-laboratory (BMI) models

**Figure 4:**
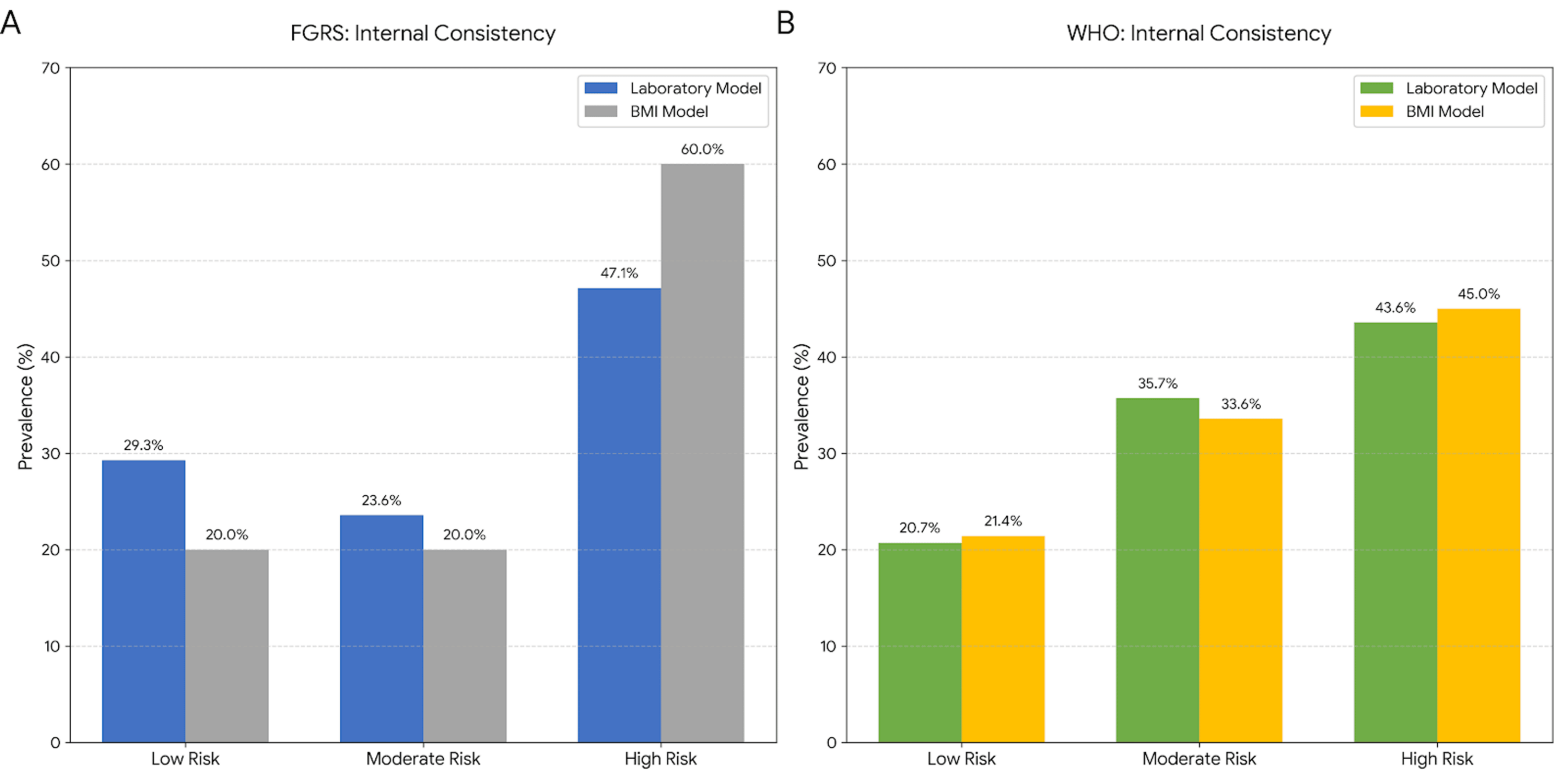
Comparison of risk prevalence between laboratory-based and BMI-based models. (A) FGRS: The comparison demonstrates high internal consistency (kappa = 0.81), with a similar risk distribution between the laboratory and BMI versions. (B) WHO: The comparison shows moderate consistency (*kappa* = 0.60) when using tool-specific thresholds. Data are presented as frequencies (%). Total sample N = 140. Significance level: *p* < 0.05. FGRS: Framingham Global Risk Score; WHO: World Health Organization Image generated using the Python programming language (v3.x), utilizing the Matplotlib and Seaborn libraries for high-resolution data visualization and statistical plotting

As a secondary objective, the study evaluated the internal consistency between the laboratory-based risk scores and their office-based (BMI) alternatives. The FGRS demonstrated high internal consistency between its two versions, achieving a weighted kappa of 0.81 (Almost Perfect agreement) for the total population (Table 2). However, as shown in Figure 4A, the substitution of cholesterol levels with BMI resulted in an increased prevalence of high-risk individuals (rising from 47.1% to 60.0%). This suggests that, despite the strong statistical agreement in ranking patients, the non-laboratory FGRS tends to overestimate absolute risk compared to the laboratory version in this population.

By contrast, the WHO charts showed lower internal consistency. Even when applying the tool-specific thresholds (≥10%), the agreement between the WHO laboratory and BMI models was classified as moderate (kappa = 0.60). While the prevalence of high-risk classification was similar in the aggregate analysis (Figure 4B), the lower kappa value indicates notable variability in individual risk assignment when laboratory data is unavailable.

## Discussion

The present study’s primary finding - that WHO charts systematically underestimate CVD risk compared to the FGRS (mean difference of 10.5% in males) - is consistent with emerging evidence from diverse global populations. Our findings align with recent studies in Asian, Caribbean, and African populations [9,12,13], which consistently reported that WHO charts yield significantly lower risk estimates compared to Framingham, suggesting a systematic calibration bias in LMIC-specific tools. Importantly, in the absence of longitudinal outcome data, this study evaluates concordance between risk estimation tools rather than their absolute predictive accuracy. This discordance is partly expected due to the divergent mathematical architectures and endpoints of the models, as FGRS predicts a broad composite of CVD while WHO focuses on fatal and non-fatal MI and stroke.

This "calibration gap" arises fundamentally from differences in the derivation cohorts. The Framingham equations were derived from prospective cohort studies with directly observed CVD events in a US population. By contrast, the WHO charts utilized relative risks combined with estimated absolute risks based on the Global Burden of Disease (GBD) modeling [14-16]. The GBD database relies on estimates that may not fully capture the elevated CVD risk in specific high-risk subpopulations, such as the present Brazilian cohort, which exhibited a 76% prevalence of metabolic syndrome [16]. Furthermore, the WHO charts for the "Americas" region encompass diverse countries, potentially diluting risk estimates for higher-risk groups within Brazil [5].

Given this discrepancy, our study applied tool-specific thresholds (FGRS ≥ 20% vs. WHO ≥ 10%) to achieve comparable risk stratification. This approach is supported by the principle that different risk scores yield systematically different absolute risk estimates [14,17]. International guidelines already demonstrate substantial variation in thresholds, ranging from 5% (SCORE) to ≥ 20% (Framingham), reflecting differences in predicted outcomes rather than disagreements about treatment benefit [17,18]. Since WHO charts systematically produce lower absolute numbers, using a lower threshold (≥ 10%) is clinically equivalent to the standard ≥ 20% Framingham threshold for initiating statin therapy [13]. Benefit-harm modeling studies confirm that such country-specific threshold adjustments are necessary to optimize prevention [13].

Regarding internal consistency, our findings support the utility of the non-laboratory (BMI-based) FGRS. The robust agreement between the laboratory and BMI-based models (kappa = 0.81) aligns with validation studies in Iran and other regions, which showed substantial to almost perfect agreement (r ≥ 0.92) between these versions [19,20]. This offers a critical advantage for the Brazilian Unified Health System (SUS): the non-laboratory model allows for immediate risk stratification in primary care settings where lipid testing may be unavailable or delayed, facilitating prompt clinical decision-making [21].

It is also important to contextualize our findings within the evolving landscape of risk prediction. While this study focused on the established FGRS and WHO tools, the American Heart Association recently introduced the PREVENT™ (Predicting Risk of cardiovascular disease EVENTs) equations [22]. Unlike the FGRS, the PREVENT equations remove race as a variable and incorporate measures of kidney function (estimated glomerular filtration rate) and optional social determinants of health metrics, reflecting a shift towards a more holistic Cardiovascular-Kidney-Metabolic (CKM) syndrome framework that includes both atherosclerotic CVD and heart failure as outcomes [22,23]. Although the PREVENT calculator was not assessed in this analysis, its emphasis on metabolic and renal factors might offer superior calibration for populations with high metabolic burden, such as our sample, although validation in Brazilian cohorts would be necessary. Future studies in Brazil should prioritize comparing the WHO charts against these newer CKM-based models to determine if they offer better discrimination and calibration than the traditional Framingham equations.

Finally, the implications for public health policy are concerning. Using the standard WHO threshold of ≥20% in our sample would classify only 11.1% of males as high-risk, compared to 59.3% by FGRS, creating a substantial "prevention gap." This could leave the majority of high-risk Brazilian men without guideline-recommended statin therapy. Given that statin use for primary prevention in LMICs is already critically low [24], our data support either recalibrating WHO charts specifically for high-risk Brazilian populations or adopting lower treatment thresholds (≥10%) to prevent widespread undertreatment. Such threshold adjustments are vital to balance the burden of preventable cardiovascular events against treatment eligibility, as highlighted in recent modeling studies [25].

This study has limitations inherent to its cross-sectional design, which prevents the assessment of causality or the verification of actual cardiovascular events over time. Consequently, our analysis relies on risk estimation surrogates rather than observed outcomes. Additionally, the sample size (n = 140) and the single-center setting in a tertiary care facility may limit the generalizability of findings to the broader primary care population. However, this high-risk profile highlights the critical need for tool calibration in specialized settings where treatment decisions have immediate prognostic implications. Therefore, the findings are more directly applicable to specialized outpatient or high-risk clinical populations rather than to the general community. The tertiary setting and the convenience sample of 140 participants characterize this study as a preliminary pilot investigation. While these factors limit generalizability, they provide a 'clinical stress test' for these tools in high-morbidity environments, serving as a catalyst for larger national trials.

## Conclusions

The WHO cardiovascular risk charts appear to underestimate cardiovascular risk in this high-risk Brazilian population when standard thresholds (≥20%) are applied, showing poor concordance with the FGRS. This discrepancy creates a significant prevention gap, potentially excluding a majority of high-risk candidates from guideline-recommended statin therapy. Our results demonstrate that adopting a tool-specific lower threshold of ≥10% for the WHO charts is required to achieve clinical equivalence with the Framingham high-risk category. In addition, the non-laboratory (BMI-based) versions of both tools showed adequate internal consistency, supporting their utility in resource-constrained settings. Therefore, strict adherence to the standard WHO thresholds (≥20%) may result in the systematic undertreatment of high-risk individuals in Brazil. To avoid this prevention gap, health policies should consider prioritizing the regional recalibration of these charts or officially adopt lower intervention thresholds (≥10%) to achieve clinical equivalence with the Framingham standard.
